# Bombay Blood Group: A Report of Two Cases

**DOI:** 10.7759/cureus.59620

**Published:** 2024-05-04

**Authors:** Asma Nasir, Aiman Minhas, Ayisha Imran, Omar Chughtai, Akhtar S Chughtai

**Affiliations:** 1 Department of Haematology and Blood Bank, Chughtai Institute of Pathology, Lahore, PAK; 2 Department of Pathology and Laboratory Medicine, Chughtai Institute of Pathology, Lahore, PAK

**Keywords:** pooled 'o' cells, anti-h lectin, rare blood group, h-antigen, bombay blood group

## Abstract

Timely detection of rare blood groups can be lifesaving, as individuals with these groups can only receive blood products from donors within the same group. The Bombay blood group is characterized by the absence of A, B, and H antigens on the surface of RBCs and can be easily missed in routine blood grouping if only forward grouping is performed. In reverse grouping, it is necessary to test the patient's serum with pooled O cells to differentiate between the O and Bombay blood groups. Further workup is conducted by testing the patient's red cells with anti-H lectin (antisera), where the absence of an agglutination reaction suggests the Bombay phenotype. In blood group O testing, the patient’s blood serum mixed with pooled O cells yields no agglutination reaction in reverse typing, whereas testing RBCs with anti-H lectin results in a strong agglutination reaction, as H-antigen is present at its highest concentration in these individuals.

Correct diagnosis of such rare blood types can save patients' lives as well as prevent the consequences of a wrong blood transfusion.

Here we present two cases that were diagnosed as having the Bombay phenotype on blood group testing in our blood bank. Both were initially misdiagnosed as blood group O by an outside laboratory.

Correct diagnosis of rare blood groups in blood banks is imperative, as a misdiagnosis can result in fatal outcomes.

## Introduction

Human blood groups have been studied since their first discovery by Karl Landsteiner in 1900 [1]. According to the International Society of Blood Transfusion, 45 different human blood group systems containing 360 red cell antigens have been identified to date. Some important blood group systems include ABO, Rh, Kell, Duffy, MNS, P, Lutheran, and Kidd. The ABO system is the most significant, as individuals older than six months typically carry clinically significant levels of antibodies, anti-A and anti-B, which target A and B antigens respectively. In contrast, blood group A contains anti-B antibodies, blood group B contains anti-A antibodies, and group O contains both anti-A and anti-B antibodies [2].

A precursor antigen to all ABO group antigens is the H antigen, which is produced by a fucosyltransferase enzyme and is subsequently converted into A or B antigens or remains unmodified in those with blood group O. Thus, H antigen reaches its highest concentration in blood group O and the lowest in blood group AB. The gene that encodes this fucosyltransferase enzyme is located on the H-locus-containing FUT1 gene, which is functional in the form of H/H or H/h. However, the h/h genotype results in the so-called 'Bombay' phenotype [3], a rare blood group first identified in 1952 by Dr. Bhende in Bombay, India, and described as the Bombay blood group. The prevalence of the Bombay blood group is about 1 in 10,000 in India and 1 in 10^6^ in Europe.

Rare blood groups, such as the Bombay blood group, pose unique challenges in blood banking due to their low prevalence and specific antigenic profiles. The Bombay group is commonly misinterpreted as group O, and individuals are often incorrectly given blood group O units in times of need. As individuals of the Bombay blood group produce clinically significant levels of anti-H antibodies, only blood products belonging to the same blood group can be administered to them. Incompatible blood transfusions lead to hemolytic transfusion reactions, which contribute to morbidity and mortality. Timely detection of such cases is crucial [4].

In this work, we report the following two cases who presented to our blood bank for blood-typing and cross-matching.

## Case presentation

Case one

A 20-year-old female delivered at full term by spontaneous vaginal delivery at a tertiary care government hospital presented to our blood bank. Her peripheral blood counts showed a hemoglobin (Hb) level of 6.6g/dL, a total leukocyte count (TLC) of 7.2 x 10^3/uL, and a platelet count of 321x10^3/uL. Her gynaecologist requested a blood transfusion with two packed cells. An outside laboratory reported her blood group as “O positive”. She was transfused with O positive packed cells. Shortly after the transfusion started the patient developed shortness of breath, and the transfusion was stopped immediately. After 24 h, the patient received another transfusion with O negative packed cells (washed RBCs). Unfortunately, after only 50 mL of transfusion, she again developed shortness of breath and swelling of her face.

Her repeat complete blood count showed a Hb of 2.8 g/dL, a TLC of 10.5x10^3 and a platelet count of 220x10^3. We received a request for blood cross-matching at our blood bank. On blood group typing, her results were graded as shown in Table 1.

**Table 1:** Forward and reverse grouping of case one. Agglutination reaction strength is graded as negative (0) / strong positive (4+)

Forward Grouping				Reverse Grouping			Auto-control
Anti-A	Anti-B	Anti-D	Anti-H lectin	A cells	B cells	O cells	0
0	0	4+	0	4+	4+	4+	

The blood group typing results were interpreted as ABO: Bombay phenotype, Rh: Positive.

A family screening of the patient showed that two of her siblings belonged to the 'O positive' blood group, whereas a 16-year-old sister and a 19-year-old brother were also found to possess the Bombay phenotype. Her 19-year-old brother was selected as a potential donor, and a crossmatch showed that he was compatible at the anti-human globulin phase of cross-matching. Eventually, the patient was transfused, and her Hb level increased to 5.3 g/dL after one transfusion.

Case two

A 35-year-old gravida 3 para 2 female was referred to our blood bank for blood grouping. She had never been transfused, and her obstetrician ordered blood grouping because she was anemic and scheduled for an elective cesarean section. She reported her blood group as O positive but did not provide any record of it.

**Figure 1:**
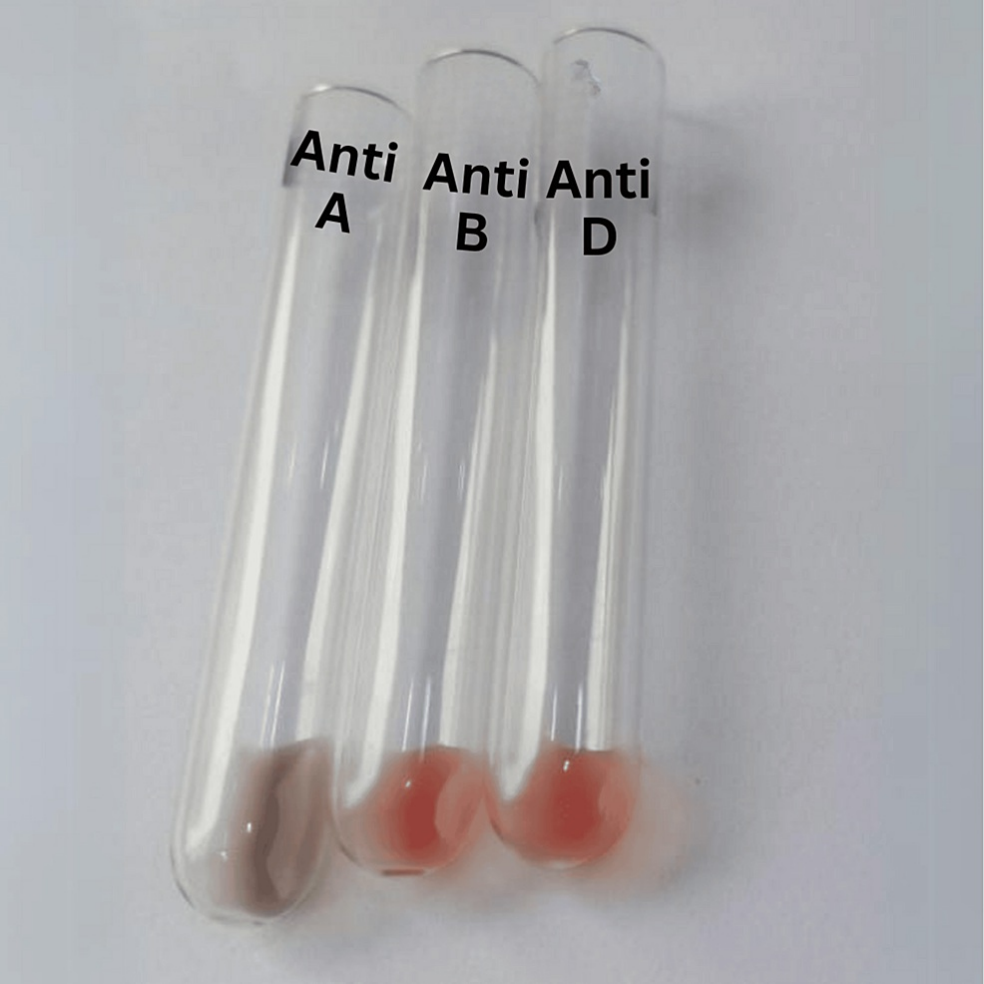
Forward blood group typing of case two.

**Figure 2:**
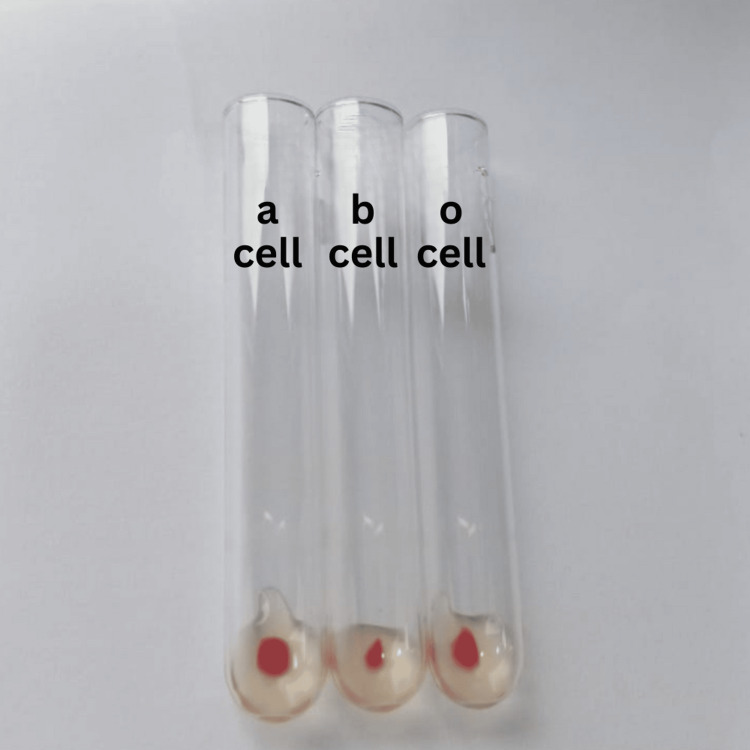
Reverse blood group typing of case two.

Figures 1 and 2 show the results of her forward and reverse blood group typing, respectively.

On blood group typing, the results were graded as shown in Table 2.

**Table 2:** Forward and reverse blood group typing of case two. Agglutination reaction strength is graded as negative (0) / strong positive (4+).

Forward group typing				Reverse group typing		
Anti-A	Anti-B	Anti-A,B	Anti-D	A	b	0
0	0	0	4+	4+	4+	4+

**Figure 3:**
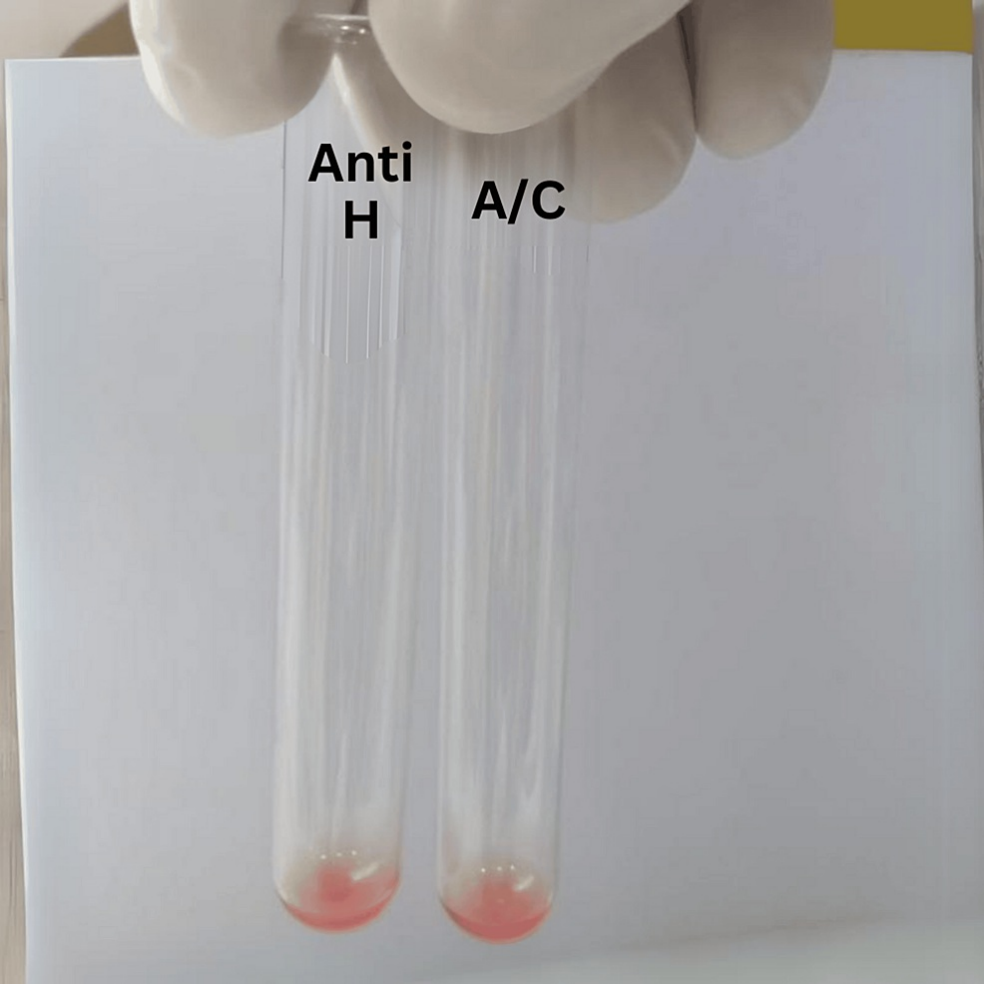
Reactions of anti-H lectin and auto control.

Figure 3 and Table 3 show further testing of the patient’s red blood cells with anti-H lectin and running auto control.

**Table 3:** Testing with auto control and anti-H lectin. Agglutination reaction strength is graded as negative (0) / strong positive (4+).

Auto control	Anti-H lectin
0	0

The blood grouping results were interpreted as Bombay phenotype, Rh positive.

Upon further history-taking, the patient reported that she had two siblings, one of whom belonged to the Bombay blood group.

## Discussion

The Bombay blood group is rare and often misinterpreted as blood group O. Individuals with blood group O have anti-A and anti-B antibodies and no anti-H [5]. In contrast, the Bombay phenotype possesses both anti-A and anti-B antibodies, and, as these individuals lack H antigens, they also have anti-H antibodies. These anti-H antibodies are naturally occurring immune globulin M (IgM) antibodies that can cause intravascular hemolysis [6]. Therefore, it is extremely important to identify this blood group because the clinically significant anti-H antibodies produced can result in an acute hemolytic transfusion reaction, which can manifest as disseminated intravascular coagulation or acute renal failure and can be fatal [7]. Moreover, these individuals can only be transfused with blood products from donors of the same blood group, and finding a donor when needed is challenging due to the rarity of this phenotype [8]. Under-reporting and non-maintenance of registries of correctly diagnosed cases also contribute to the lack of availability of suitable donors.

Various approaches have been adopted to manage these patients when blood from Bombay donors is not available, including the use of autologous transfusion, fresh frozen plasma, crystalloids, and colloids [4]. Another approach is acute normovolemic hemodilution, in which controlled removal of whole blood from the patient is performed immediately before a surgical procedure, followed by the administration of colloid or crystalloid solutions to maintain normal blood volume with a reduced red cell mass. The collected whole blood is then re-infused after the procedure inside the operation theater [9]. One study described the successful use of acute normovolemic hemodilution during an emergency cesarean section in a pregnant female with the Bombay phenotype [10]. Another study reported the management of a gravida patient with bleeding who underwent dilatation and curettage by the administration of colloids and crystalloid; however, no autologous blood transfusion was given [11]. Pre-operative autologous donation is a reasonable approach in diagnosed cases of the Bombay group for which elective procedures are planned, wherein repeated donations stimulate bone marrow erythrocyte production [12]. Another approach is intraoperative cell salvage, in which blood spilled in the surgical field is collected and re-infused into the patient. However, this method can only be implemented if automated cell salvage devices are available, but it is applicable for both elective and emergency surgical procedures [13].

Based on this case series, we recommend that government transfusion services operating in regions where the Bombay blood group is prevalent maintain a registry of such individuals, as it could aid in increasing donor availability in times of need. Moreover, family members of such individuals should also be tested to determine whether they have the same blood group. The cases we received at our blood bank were misinterpreted by outside laboratories as blood group O. One of our patients (case one) experienced an acute hemolytic transfusion reaction with a significant decrease in Hb; whereas the other case (case two) had fortunately never been transfused, so no significant morbidity occurred.

## Conclusions

As the Bombay blood group phenotype is commonly misinterpreted as blood group O, it is crucial to include testing with 'O' cells in reverse blood grouping in routine blood banking. In all discrepant cases, testing with anti-H lectin and running auto control can lead to the correct diagnosis, which can prevent clinical consequences during routine testing. However, in emergency settings where administration of O-negative units may lead to a subsequent hemolytic transfusion reaction, the differential diagnosis of rare blood groups should always be considered.
